# The influence of visual supports and motivation on motor performance of the MABC-2 for Chinese school-aged children with autism spectrum disorder

**DOI:** 10.1038/s41598-021-95155-8

**Published:** 2021-07-30

**Authors:** Xiaoyi Hu, Hui Wang, Zhuo Rachel Han, Yu Zhao, Li Ke

**Affiliations:** 1 School of Special Education, Education Research Center for Children with Autism, Faculty of Education, Beijing Normal University, Beijing, China; 2 School of Artificial Intelligence, Faculty of Psychology, Beijing Normal University, No. 19 Xinjiekou Outer Street, Beijing, 100875 China; 3 Beijing Rainbow Town Rehabilitation Center, Beijing, China; 4 Collaborative Innovation Center of Assessment for Basic Education Quality, Beijing Normal University, Beijing, China

**Keywords:** Human behaviour, Rehabilitation

## Abstract

The influences of including visual supports and strategies to increase motivation for children with autism spectrum disorder (ASD) in motor assessments were examined. 97 children with ASD and 117 age-matched typically developing (TD) children performed the Movement Assessment Battery for Children, Second Edition (MABC-2) under traditional, visual support, motivation, and visual support plus motivation protocols. Results showed that children with ASD elicited lower MABC-2 scores than TD children. Moreover, in children with ASD, the visual support protocol, but not the motivation protocol, produced higher scores on ball and balance skills than the traditional protocol. These findings indicated that children with ASD were developmentally delayed in motor skills; however, their performance may be improved by including visual supports in motor assessments.

## Introduction

Autism spectrum disorder (ASD) is a neurodevelopmental disorder characterized by social communication deficits and restricted and repetitive behaviors^1^. Empirical evidence has consistently demonstrated that in addition to deficits in social communication skills and stereotyped behaviors, children with ASD exhibit significant delays in motor skill development compared to their typically developing (TD) peers^2–4^. Delays and deficits in motor skill development are indeed a core feature of the ASD phenotype^5^, thus the administration of motor skills assessments may facilitate screening for motor delays and then detecting of ASD. However, these assessments are primarily conducted in high-functioning youth and adults with ASD or in those with Asperger syndrome^6–9^. The limited numbers of studies including participates with low-functioning ASD or moderate to severe ASD is possibly the result of this population having tremendous challenges related to verbal instruction comprehension and lack of motivation to engage in assessment. Moreover, due to the impairments in gross and fine motor skills and motor skill proficiency, individuals with ASD demonstrates clear symptoms of developmental coordination disorder (DCD)^10,11^. DCD is a chronic neurological disorder featured by impairments in movement planning and coordination^1^. In a systematic review of 11 studies targeting populations of individuals with ASD and DCD, Caçola et al. concluded that individuals with ASD and DCD shared similar deficits in motor skills^12^.

The Movement Assessment Battery for Children (MABC and MABC-2), a standardized test for DCD, assesses manual dexterity, ball skills, and balance skills used in daily activities in children 3–16 years old^13,14^. This assessment has been used more frequently to evaluate motor development in children with ASD^7,15,16^. Ament et al. compared the MABC-2 performance scores of 56 children with ASD to 63 aged-matched children with ADHD and 81 TD children^17^. The MABC-2 scores revealed that both fine and gross levels of motor performance were significantly lower in children with ASD than in their TD and ADHD peers, especially in catching tasks and balancing tasks requiring visual and temporal feedback to guide or balance movement. In related research, Liu and Breslin found that 77% of 30 children with ASD were clearly delayed in their motor skill performance when compared to that of age-matched TD children^7^.

However, studies have found that the poor motor performance exhibited by children with ASD may not exclusively be due to impaired motor skills but rather difficulties in understanding task specifications^4,18,19^. The MABC-2 manual encourages examiners to use verbal instructions and physical demonstrations to ensure children understand mandatory tasks^20^. Children with ASD have difficulties in processing auditory instructions or verbal directions and understanding body language delivered by the instructors^21,22^. Consequently, performance on the MABC-2 in children with ASD may be compromised.

Researchers have reported that the use of visual supports with traditional motor skill testing batteries can be one possible adaptation strategy to truly assess motor skill development in children with ASD because these children can process and interpret visual stimuli more easily than auditory information^23,24^. As one evidence-based intervention strategy in general ASD education practices and research, visual supports, including picture task cards, picture activity schedules, and visual prompts, are effective for developing flexibility and independence, improving social interactions and communications, and decreasing off-task behaviors^25,26^.

The picture activity schedule, a visual schedule presenting a series of activities to be completed in a certain order, can be realized via pictures, photos, and line drawings^27^. Due to the concrete, relevant information provided through pictures, children with ASD are more likely to understand abstract concepts, process verbal directions, and organize their actions independently^23^. During the last decade, researchers have adopted picture activity schedules to help measure the motor skills of children with ASD^22,25^. For example, Liu and Breslin reported that children with ASD scored higher on the MABC-2 when given a picture activity schedule^28^. Furthermore, Allen and colleagues (2017) illustrated that a visualized Test of Gross Motor Development-3 (TGMD-3) protocol could be a valid and reliable assessment of gross motor performance for children with ASD by facilitating their task analyses and comprehension^21^. These studies have provided substantial evidence for adapting traditional motor assessments to improve the comprehension of tasks and further facilitate optimal motor performance of children with ASD.

In addition to the comprehension issue, attention and effort issues within assessments should be addressed^29^. A basic assumption of the motor skill assessments is that the participants are full involved and devote the maximum level of energy and effort to complete every single task^18^. However, compared to TD children, children with ASD are more likely to be unmotivated or show a limited range of interest in assessment activities^29–31^. Lack of motivation during assessments could inaccurately capture motor abilities of children with ASD and, therefore, could yield an underestimation. Therefore, it is necessary to contrive motivation variables to establish behavioral compliance and increase task engagement in children with ASD during these assessments.

One evidence-based strategy used to contrive motivation in children with ASD is behavioral momentum. Behavioral momentum, also known as a high-probability command sequence, entails making 3 to 5 requests that are easy for a child to perform before making a request that is more challenging or difficult to perform^32^. By following this pattern of easy tasks, the instructors can efficiently motivate children with ASD to be compliant or engage in task completion. Although no previous studies have incorporated strategies to increase motivation into motor assessments, behavioral momentum has been found to be effective in increasing compliance^33,34^, social interactions with peers^35^, and positive behavioral fluency outcomes in children with ASD^36^.

In China, the estimated prevalence of ASD has increased to 1% of the total population, accounting for 13 million people^37^. The increasing prevalence of ASD has made the use of motor skill development measurements high priorities for researchers and practitioners in China to understand overall motor development of children with ASD and design, as well as implement, targeted intervention practices. However, research examining motor skills in Chinese children with ASD is still limited.

Therefore, the present study provides an initial examination of motor skill development in Chinese school-aged children with moderate to severe ASD utilizing the MABC-2. Specifically, this study aims to investigate motor skill performance in children 7–10 years with ASD and in age-matched TD children. It was hypothesized that children with ASD would display lower scores in overall and three domains of the MABC-2 compared to their age-matched TD children. Moreover, this study measured motor performance of children with ASD via the MABC-2 under four assessment conditions: (a) the MABC-2 traditional protocol, (b) the MABC-2 visual support protocol, (c) the MABC-2 motivation protocol, and (d) the MABC-2 visual support plus motivation protocol. We hypothesized that children with ASD would benefit from incorporating visual support and strategies to increase motivation and, therefore, would demonstrate higher scores under visual support and motivation protocol conditions than those children using traditional protocol.

## Results

### Descriptive analyses

Descriptive statistics for MABC-2 scaled scores revealed that almost all children with ASD (99.0%, 96 children with ASD) were identified as having a significant DCD and 1.0% (1 child with ASD) was identified as at-risk for a DCD. In contrast, 86.3% of TD children (101 TD children) did not have a DCD, 6.0% (7 TD children) were at risk for a DCD, and the remaining 7.7% (9 TD children) had a significant DCD.

### Group differences between children with ASD and TD children on the MABC-2

The first goal of the study was to examine the motor performance in children with and without ASD. An independent sample *t*-test revealed a significant group difference in total scaled scores (*t* (212) = − 30.20, *p* < 0.001), with children with ASD, regardless of which protocol was used, having lower total scores than their age-matched TD peers. In addition, a 2 (group: ASD, TD) × 3 (subscale: manual dexterity, aiming and catching, and balance) mixed-design analysis of variance (ANOVA), with group as a between-subjects variable and subscale as a within-subjects variable, was performed on the scaled scores of the MABC-2. Significant main effects were found for group (*F* (1, 212) = 1013.22, *p* < 0.001, *η*^2^ = 0.83) and subscale (*F* (2, 424) = 34.71, *p* < 0.001, *η*^2^ = 0.14). There was also a significant interaction between group and subscale (*F* (2, 424) = 20.83, *p* < 0.001, *η*^2^ = 0.09). Post hoc tests showed that compared with TD children, children with ASD had lower scaled scores on manual dexterity (*p* < 0.001; mean difference = − 8.64, 95% confidence interval [CI] = [− 9.29, − 8.00]), aiming and catching (*p* < 0.001; mean difference = − 6.14, 95% CI = [− 6.89, − 5.39]), and balance (*p* < 0.001; mean difference = − 8.13, 95% CI = [− 8.71, − 7.55]). Group differences in the MABC-2 scaled scores are shown in Table 1.

**Table 1:** Means and Standard Deviation of the MABC-2 scaled scores by group.

	Children with ASD		TD Children	
	*M*	*SD*	*M*	*SD*
Manual dexterity	1.34	1.29	9.98	3.01
Aiming and catching	4.09	2.53	10.23	2.98
Balance	1.64	1.75	9.77	2.43
Total score	1.16	0.69	9.76	2.73

### Performance differences within children with ASD by protocol

A 4 (protocol: traditional, visual support, motivation, and visual support plus motivation) × 3 (subscale: manual dexterity, aiming and catching, and balance) mixed-design ANOVA, with protocol as a between-subjects variable and subscale as a within-subjects variable, was conducted on the scaled scores of the MABC-2. The ANOVA revealed significant main effects of protocol (*F* (3, 93) = 4.38, *p* = 0.006, *η*^2^ = 0.12) and subscale (*F* (2, 186) = 79.81, *p* < 0.001, *η*^2^ = 0.46). There was also a marginally significant interaction between protocol and subscale (*F* (6, 186) = 5.42, *p* = 0.08, *η*^2^ = 0.06). Post hoc tests demonstrated that the aiming and catching scaled scores in the visual support protocol were higher than those scores in the traditional protocol (mean difference = 1.95, *p* = 0.005) and the motivation protocol (mean difference = 1.97, *p* = 0.008); the balance scaled scores in the visual support protocol were higher than those scores in the traditional protocol (mean difference = 1.14, *p* = 0.019) and the motivation protocol (mean difference = 1.28, *p* = 0.014); however, the manual dexterity scaled scores of children with ASD did not differ by protocol. Finally, a univariate ANOVA was conducted on the total scaled scores of children with ASD in each of the four protocols. No significant main effect of the total scaled scores was found. Table 2 depicts the mean and standard deviation for scaled scores on the MABC-2 for all studied protocols.

**Table 2:** Means and standard deviations for ASD children’s scaled scores on the MABC-2 for each protocol.

Motor skills	TP (n = 30)		VSP (n = 22)		MP (n = 23)		VS + MP (n = 22)	
	*M*	*SD*	*M*	*SD*	*M*	*SD*	*M*	*SD*
Manual dexterity	1.17	0.65	1.27	0.94	1.39	1.88	1.59	1.53
Aiming and catching	3.37	1.88	5.32	2.85	3.35	2.35	4.64	2.66
Balance	1.07	0.37	2.41	2.48	1.13	0.63	1.91	2.00
Total	1.07	0.37	1.18	0.50	1.22	1.04	1.23	0.75

*TP* traditional protocol, *VSP* visual support protocol, *MP* motivation protocol, *VS* + *MP* visual support plus motivation protocol.

## Discussion

The purpose of this study was to empirically examine motor skill performance in Chinese children with ASD and their age-matched TD peers and to further explore the effects of visual supports and motivation implemented along with the MABC-2. As hypothesized, children with ASD exhibited pronounced motor delays compared to TD children based on their overall performance on the MABC-2 regardless of how instructions were provided. In addition, significant interaction was found between group and subscale, suggesting that the ASD group exhibited lower scores on all three components (i.e., manual dexterity, aiming and catching, and balance skills) than the TD group. These findings are generally consistent with the existing literature, whereby children with ASD display developmental delays in fine and gross motor abilities^3,4,38^. Given the prevalence of motor impairments, studies have reported that 42–79% of children with high-functioning ASD or Asperger syndrome experience definite motor deficits^7,39,40^. In our sample, almost all children with moderate to severe autism (99%) were found to have delayed motor development. Indeed, motor impairments have been found to be related to autism severity^39,41^. The current results also extend previous findings revealing that motor impairments were more common or more severe in children with low-functioning or moderate to severe ASD^7,16^.

The main hypothesis proposed that including picture activity schedules and strategies to increase motivation in the MABC-2 would elicit higher MABC-2 scores in children with ASD than the traditional protocol. This hypothesis was partially supported. Specifically, significant interaction effect between protocol and subscale was found within children with ASD, revealing that visual support protocol elicited higher scores on aiming and catching and balance constructs than the traditional protocol. The incorporation of picture activity schedules can help to direct attention and facilitate task understanding in children with ASD, which, in turn, can help to successfully complete the motor assessment, especially in tasks evaluating ball skills and balance skills. This finding is in line with previous studies showing that adaptations to motor skill assessments that incorporate visual supports can maximize performance in children with ASD and result in higher scores on assessments^21,23,25,28^. Furthermore, our results corroborate previous views that children with ASD are often visual learners; they can process and interpret visual information more effectively than auditory information^24,42^. To accurately evaluate motor performance, it may be beneficial for children with ASD to perform assessments with additional visual supports to improve the comprehension of the assessment. It is noteworthy that significant interaction effect was only found in the subscales of aiming and catching and balance, suggesting that visual supports can help children with ASD achieve higher scores on ball skills and balance skills rather than manual dexterity. Most of the children with ASD included in our study were from special education schools in mainland China. These schools provide training sessions with Special Olympics and adaptive physical education (APE), affording more opportunities to practice ball skills and balance skills. As performance scores determined by motor assessments rely heavily on previous experience^4,14^, children with ASD in our sample might have experienced less severe ball skill and balance skill impairments and might have been more likely to benefit from visual supports when evaluating these skills. In contrast, there was no significant difference in the manual dexterity scores of children with ASD in different assessment protocols. It may be that manual dexterity is the more severely affected area of motor skills among children with ASD, thus, even with modified instructions, it is difficult to improve performance on this construct. Indeed, our results indicated that regardless of the protocols used, the weakest scores in children with ASD in our sample were manual dexterity, with little variability. Related studies have shown mixed results on specific areas of motor impairments in children with ASD. Some studies found that children with ASD displayed more deficits in ball skills^17,43^, whereas other studies reported a more common occurrence of deficits in manual dexterity^40,41,44^. Our results lend support to the latter notion that children with moderate to severe autism might exhibit more severe deficits in manual dexterity, and they may not benefit as much from visual supports when performing this skill. The findings highlight the importance of examining specific motor components to further clarify differences in motor skills components, as well as the effectiveness of adaptation strategies in motor assessments.

Another possible explanation for this finding is that the incorporation of picture activity schedules into the administration of the manual dexterity skill assessment increases the time needed to complete the tasks, resulting in lower performance scores in children with ASD. In the manual dexterity domain of the MABC-2, two of the three tasks (i.e., placing pegs and threading lace) require the coupling of speed and accuracy. Although facilitating comprehension, the inclusion of visual supports in the assessment may extend the time needed to complete the tasks. Breslin and Rudisill explored differences in the total amount of time needed to complete the TGMD-2 assessment in children with ASD for each of the three protocols (i.e., traditional protocol, picture task card protocol, and picture activity schedule protocol)^45^. The results showed that the picture activity schedule protocol took a significantly longer amount of time to complete than the other two protocols. Finally, the assessment tools used in the MABC-2 kit might influence the performance scores of children with ASD. It was commonly observed that children with ASD in our sample showed perseverative interest in the assessment tools, especially those used to evaluate their manual dexterity skills, such as the yellow pegs, blue peg board, and red lace. They could become overly excited about the colorful assessment tools and devoted their attention to these tools instead of the picture activity schedules or the motor performance tasks. Distraction due to restricted interests, a core characteristic of ASD, may help to explain why children with ASD received lower manual dexterity scores and did not benefit as much from the addition of visual supports into the assessment.

This study also sought to explore the influence of motivation on motor performance in the MABC-2. In contrast to our hypothesis, neither the overall score nor the scores of the three subscales of children with ASD have improved by including strategies to increase motivation. Researchers have noted that attention, comprehension, and effort are prerequisite assumptions of all motor assessments, and all these issues should be addressed to generate accurate evaluation^46^. When working with children with ASD, most research has focused on modifications such as visual supports to facilitate children’s comprehension^21,25,28^. However, without participants’ full engagement in each task, the assessment results may not capture their true abilities^47,48^. The current study, to our knowledge, was the first to increase on-task behaviors of children with ASD by adding a strategy to increase motivation (i.e., behavior momentum). Nonetheless, no significant improvement in performance scores was found in the motivation protocol compared to the traditional protocol.

Consistent with previous studies^33,49^, we adapted six high-probability behaviors, such as ‘high five’ and ‘hands up’, and provided verbal praise as reinforcement. However, these high-probability commands might have been too easy to increase children’s motivation to participate in the following tasks that were included in the MABC-2. It may also due to the fact that for many children with moderate to severe ASD, the verbal praise provided contingent on task completion did not function as an effective reinforcer and, consequently, it did not improve motor performance^50^. Thus, more powerful reinforcers may be needed to increase the motor performance of children with ASD. Finally, this nonsignificant finding can be attributed to the arrangement of the high-probability command sequences used to motivate the engagement of children with ASD. To avoid fatigue and exhaustion in the children, we only added behavioral momentum once prior to proceeding with the whole MABC-2 assessment. The typical assessment time of the MABC-2 is 20–30 mins^45^, whereas in our sample with moderate to severe autism, it usually took the child more than one hour to complete all tasks. To be more efficacious, high-probability requests should be immediately followed by the target behaviors^36,49^. For a lengthy assessment duration, the positive influence of the reinforcement might be weakened at the end of the assessment if it is only included once.

There are several limitations of this study that should be noted when interpreting our findings. First, our sample was restricted to 7–10-year-old children with ASD and most of them with moderate to severe ASD symptoms, limiting the generalizability of our findings to the entire spectrum of ASD. More studies with wider ranges of age and ASD severity are encouraged to replicate the current findings. Second, the cognitive function of participants was not assessed in this study. IQ has been shown to correlate with motor skill performance of individuals with ASD, even in high functioning samples^51^. In this study, we tried to mitigate the impact of IQ on the results, including recruiting ASD children with an IQ lower than 70 to make their cognitive levels relatively homogeneous, and randomly assigning them to different protocols. Future research is recommended to assess and control for IQ to better understand genuine improvement in motor performance scores with the inclusion of visual supports and strategies to increase motivation. Finally, as mentioned above, behavioral momentum was only added once prior to formal MABC-2 administration, which might compromise the positive effects of motivation on task participation. Increasing the overall rate of high-probability commands so that children can receive reinforcement more frequently in future studies would help verify the influence of reinforcement rates on children’s motor performance. Moreover, other motivational strategies, such as providing choice and autonomy^52^, could also be considered in future research to elicit intrinsic motivation and, therefore, task engagement in children.

Almost all children with ASD in our study, regardless of the protocol provided, displayed pronounced motor impairments in the overall MABC-2 scores and all three domains compared to age-matched TD peers. Our results provide empirical evidence to support the idea that children with ASD are developmentally delayed in terms of motor skill development. Fine and gross motor skills are fundamental skills serving as the basic building blocks for more complex movements^53^. In addition, motor proficiency may exert an influence on other areas of development, such as cognitive function, social communication, and physical health^3,47,54^. Therefore, it is paramount to provide movement-based interventions for children with ASD to improve their overall functioning.

Moreover, children with ASD had higher scores on the aiming and catching and balance domains of the MABC-2 in the visual support protocol than in the traditional protocol. It seems that by capitalizing their relative strengths in processing visual information, visual supports can help to facilitate understanding and, therefore, elicit higher performance scores^21,25,28^. Our study emphasizes the need for adaptation strategies to drive true levels of motor performance of children with ASD because accurate motor assessments provide valuable information for early interventions. We highly recommend that researchers and practitioners incorporate visual supports, such as picture activity schedules, when evaluating motor skills of children with ASD.

In conclusion, the findings of this study demonstrated that children with moderate to severe ASD exhibited deficits in fine and gross motor skills on overall MABC-2 assessment scores, as well as all three of its constructs, compared to age-matched TD peers. Moreover, including picture activity schedules in the visual support protocol elicited higher scores on ball skills and balance skills than the traditional protocol. However, no significant improvement was observed in the protocol incorporating behavioral momentum to increase motivation. Our results provide further evidence suggesting that children with ASD may benefit from the inclusion of visual supports in motor skill assessments.

## Methods

### Participants

A total of 234 children aged 7–10 years old participated in the present study, including 117 children with ASD and 117 TD children. Children with ASD were recruited from advertisements distributed to 11 public special education schools serving children with ASD and 8 regular schools in Beijing, China. Children with ASD were originally diagnosed by a psychiatrist according to the Diagnostic and Statistical Manual of Mental Disorders, Fifth Edition (DSM-V)^1^. Diagnoses were further confirmed with the Chinese version of the Childhood Autism Rating Scale, Second Edition (CARS-2)^55,56^ by a licensed psychologist. The total scores of the CARS-2 classify children as having severe (above 36.5), mild to moderate (30–36.6), or minimal to no autism (15–29.5). With respect to cognitive abilities, all children with ASD met the admission criteria of a full-scale IQ < 70 on the Wechsler Abbreviated Scale of Intelligence (WASI)^57^ in order to receive special education services provided by schools in the city where information was collected. Thus, children with ASD had cognitive delays, although we did not assess their specific IQs in the current study. TD children were recruited from public elementary schools and were matched to the children with ASD on age, *t* (212) = 0.35, *p* > 0.05, and sex, *t* (212) = − 0.44, *p* > 0.05.

Ten children with ASD were excluded from the data analyses due to incomplete data sets. The excluded and included participants did not differ in any demographic characteristics. All TD children met the inclusion criteria and completed all tasks of the MABC-2. Thus, the final sample consisted of 97 children with ASD (*M* age = 8.52 years, *SD* = 1.05 years; 81 boys and 16 girls) and 117 TD children (*M* age = 8.47 years, *SD* = 1.05 years; 95 boys and 22 girls). Based on the CARS-2 scoring criteria, 76 children with ASD (78.35%) demonstrated severe autism, and 21 (21.65%) demonstrated mild to moderate autism (range = 31–55, *M* = 41.66, *SD* = 5.80).

### Measures

The Movement Assessment Battery for Children, Second Edition (MABC-2)^14^, a performance-based assessment, was used to assess children’s gross and fine motor skills. The MABC-2 contains 8 individual tests measuring 3 dimensions of motor impairments: manual dexterity (MD), aiming and catching (AC), and balance (BAL). The individual tests of the MABC-2 are divided into three age bands (3–6 years, 7–10 years, and 11–16 years), with children undertaking different tasks depending on their age. For the present sample, the second age band was administered. The MD dimension includes 3 tasks: placing pegs, threading lace, and drawing trails. The AC dimension includes 2 tasks: catching with two hands and throwing a beanbag onto a mat. The BAL dimension includes 3 tasks: one-board balance, walking forwards heel-to-toe, and hopping onto mats.

Raw scores of each task are converted to four scaled scores (i.e., three for subscale scores and one for total score) based on the Chinese normative sample^58^. The converted scaled scores range from 1 to 19 (*M* = 10; *SD* = 3), with lower scores representing a higher severity of motor skill impairments. Moreover, the scaled total scores of the MABC-2 can be used to identify children with significant (5 or less), at-risk (6), or no motor coordination difficulties (7 or more).

### Procedures

All study procedures were approved by the Institutional Review Board of the Beijing Normal University, China. All children and their parents signed the informed consent form before each child’s participation. The primary investigator and two research assistants administered all tasks of the MABC-2 in a quiet room at each of the local schools. The investigator familiarized participants with the assessment environment and procedures. Children were tested individually and their movement performance was videotaped. The principal investigator evaluated the children’s motor skills based on each child’s video-recorded performance on every task for each protocol of the test.

All TD children were asked to complete the MABC-2 using the traditional protocol. To examine whether including visual supports and behavioral momentum in the MABC-2 assessment would benefit children with ASD, four MABC-2 assessment protocols (i.e., traditional, visual support, motivation, and visual support plus motivation protocols) were administered to children with ASD. Each child with ASD was randomly assigned to complete one protocol of the MABC-2. This resulting in 30 children with ASD were assigned to the traditional protocol, 22 to the visual support protocol, 23 to the motivation protocol, and 22 to the visual support plus motivation protocol. Moreover, children with ASD in different protocols had no significant differences in the severity of ASD symptoms based on the CARS-2 classification (*F* (3, 93) = 0.58, *p* > 0.05).

In the traditional protocol condition, each child received detailed verbal instructions and physical demonstrations prior to motor skill performance. They were also provided with additional descriptions and demonstrations if the child did not seem to understand the task or if he or she verbally requested to see the skill again.

During the visual support protocol, a picture activity schedule of each task was provided to the child before he or she performed each motor skill. The picture activity schedule presented a sequence of activities (e.g., placing pegs, catching with two hands) that should be performed in a temporal sequence so the predictability of the daily schedule increased and the child with ASD could monitor the completion of each activity on the schedule. Children were told that they could look at the picture activity schedule while performing the motor skill. In this condition, verbal instructions were minimized to emphasize visual supports.

During the motivation protocol, the primary investigator randomly selected 3 of 6 high-probability behaviors (i.e., “hands up,” “high five,” “stand on one leg,” “pick up the pen on the table and give it to the teacher,” “hands up and high five the teacher,” and “stand on one leg and jump”). The investigator then presented these high-probability behaviors immediately followed by a low probability behavior (i.e., a motor task according to the MABC-2). When the child successfully demonstrated the required behaviors, the investigator provided verbal praise (e.g., “Good job, you are right”). However, if the child showed noncompliant behaviors, the investigator repeated the sequence of 3 high-probability behaviors before asking him or her to perform motor skill tasks included in the MABC-2.

During the visual support plus motivation protocol, the investigator presented the child two high-probability behaviors prior to asking the child to display motor skills based on the MABC-2. Moreover, a picture activity schedule was shown to the children before they performed each motor skill.

### Data analyses

Raw scores of each task were converted to scaled scores, which were then summed to produce scaled scores for three subscales and one total scaled score. Descriptive analyses were performed to determine the average levels of motor skills in children with ASD and TD children. Additionally, an independent sample *t*-test and a 2 (group: ASD, TD) × 3 (subscale: manual dexterity, aiming and catching, and balance) mixed-design ANOVA were used to understand differences in total scores and subscale scores between children with ASD and TD children, respectively. Finally, to examine whether children with ASD would benefit from including visual supports and strategies to increase motivation during the MABC-2 assessment, a 4 (protocol: traditional, visual support, motivation, and visual support plus motivation) × 3 (subscale: manual dexterity, aiming and catching, and balance) mixed-design ANOVA was conducted on scaled scores of children with ASD for the four assessment protocols. Bonferroni correction was conducted as needed to adjust for multiple comparisons. Effect sizes were assessed as eta-squared (*η*^2^). All statistical analyses were performed using SPSS 21.0.

### Informed consent

Informed consent was obtained from all individual participants included in the study.

### Compliance with ethical standards

All procedures performed in studies involving human participants were in accordance with the ethical standards of the institutional and/or national research committee and with the 1964 Helsinki declaration and its later amendments or comparable ethical standards.

## Data Availability

The data that support the findings of this study are available from the corresponding author upon reasonable request.
